# Implementation of maternal and perinatal death surveillance and response and related death review interventions in humanitarian settings: A scoping review

**DOI:** 10.7189/jogh.14.04133

**Published:** 2024-07-12

**Authors:** Meighan Mary, Hannah Tappis, Elaine Scudder, Andreea A Creanga

**Affiliations:** 1 International Health Department, Johns Hopkins Bloomberg School of Public Health, Baltimore, Maryland, USA; 2 International Center for Maternal and Newborn Health, Johns Hopkins Bloomberg School of Public Health, Baltimore, Maryland, USA; 3 Center for Humanitarian Health, Johns Hopkins University, Baltimore, Maryland, USA; 4 Jhpiego, Baltimore Maryland, USA; 5 International Rescue Committee, Washington DC, USA; 6 Department of Gynecology and Obstetrics, Johns Hopkins School of Medicine, Baltimore, Maryland, USA

## Abstract

**Background:**

The global population impacted by humanitarian crises continues to break records each year, leaving strained and fractured health systems reliant upon humanitarian assistance in more than 60 countries. Yet little is known about implementation of maternal and perinatal death surveillance and response (MPDSR) within crisis-affected contexts. This scoping review aimed to synthesise evidence on the implementation of MPDSR and related death review interventions in humanitarian settings.

**Methods:**

We searched for peer-reviewed and grey literature in English and French published in 2016–22 that reported on MPDSR and related death review interventions within humanitarian settings. We screened and reviewed 1405 records, among which we identified 25 peer-reviewed articles and 11 reports. We then used content and thematic analysis to understand the adoption, appropriateness, fidelity, penetration, and sustainability of these interventions.

**Results:**

Across the 36 records, 33 unique programmes reported on 37 interventions within humanitarian contexts in 27 countries, representing 69% of the countries with a 2023 United Nations humanitarian appeal. Most identified programmes focussed on maternal death interventions; were in the pilot or early-mid implementation phases (1–5 years); and had limited integration within health systems. While we identified substantive documentation of MPDSR and related death review interventions, extensive gaps in evidence remain pertaining to the adoption, fidelity, penetration, and sustainability of these interventions. Across humanitarian contexts, implementation was influenced by severe resource limitations, variable leadership, pervasive blame culture, and mistrust within communities.

**Conclusions:**

Emergent MPDSR implementation dynamics show a complex interplay between humanitarian actors, communities, and health systems, worthy of in-depth investigation. Future mixed methods research evaluating the gamut of identified MPDSR programmes in humanitarian contexts will greatly bolster the evidence base. Investment in comparative health systems research to understand how best to adapt MPDSR and related death review interventions to humanitarian contexts is a crucial next step.

Maternal and perinatal death surveillance and response (MPDSR) provides an opportunity for stakeholders to understand the circumstances surrounding maternal and perinatal deaths in order to improve quality of care and, ultimately, to prevent future mortality. The World Health Organization (WHO) describes MPDSR as a cyclical process of identification, reporting, and review of deaths, and an action-oriented response to address identified social and health system contributors [1]. It is a product of evolving guidance from siloed maternal death audits and reviews [2], expanded death surveillance and response systems [3], and (more recently) audit and review guidelines for stillbirths and neonatal deaths [4]. With global advocacy galvanising MPDSR as a key strategy to achieve global maternal and neonatal mortality reduction targets by 2030 [5–8], many low- and middle- income countries (LMICs) have adopted national policies and guidelines to support the implementation of MPDSR and related death review interventions [9]. However, without considering adaptations needed in humanitarian settings [10], the potential for MPDSR to impact global maternal and perinatal mortality will fall short.

A record-breaking 339 million people were in need of humanitarian assistance in 2022, with women and children being among the most vulnerable [10]. From armed conflict to environmental disasters, humanitarian settings differ widely and can affect health systems in a myriad of ways, including through collapse of infrastructure, disruption of health services, and shortages in financial and human resources, essential medicines, and supplies. In the first six months after the onset of a crisis, humanitarian health response typically prioritises the delivery of immediate lifesaving care rather than systems investments. However, with more crises becoming protracted and cyclical [11], new strategies are needed to ensure that the women and children spending decades living in these settings can receive high-quality services [12].

WHO guidelines on MPDSR [1] stress the need for MPDSR implementation in alignment with the minimal initial services package for reproductive health in crisis situations (MISP) [13], recommending implementing MPDSR alongside efforts to strengthen health systems and improve quality of care only in stabilised or protracted crises [1]. However, recommendations for how to adapt interventions to make them fit for these contexts are weak, derived from an ad hoc compilation of stakeholder perspectives [14] and a few published programmatic experiences in humanitarian contexts [14–27].

While much can be learned from prior literature reviews, including studies on implementation factors influencing MPDSR [28–32], learnings from varying humanitarian contexts could lend insights on how to optimally implement MPDSR in disrupted environments, weakened health systems, and complex governance and stakeholder landscapes. Thus, we undertook a scoping review of peer-reviewed and grey literature to synthesise evidence and glean lessons on the implementation of MPDSR and related death review interventions in humanitarian settings.

## METHODS

The scoping review was guided by the PRISMA-ScR checklist [33] and followed Arksey and colleagues’ five-step framework [34], as well as an established protocol registered with the Open Science Framework on 30 July 2021 [35].

**Figure 1:**
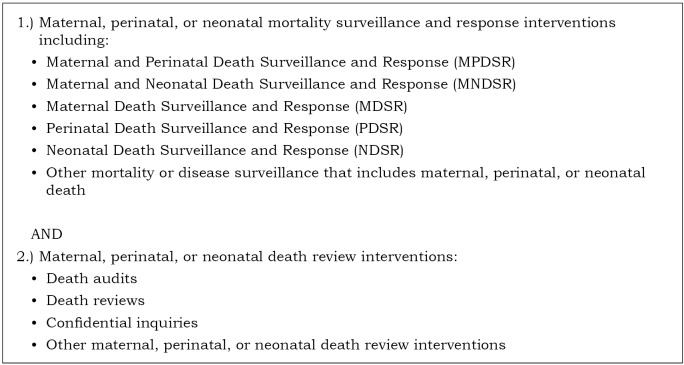
MPDSR and related death review interventions.

The primary research question for the scoping review was: ‘What is known in the literature about the implementation of MPDSR and related death review interventions in humanitarian settings?’ The interventions included maternal, perinatal, or neonatal mortality surveillance and response systems and maternal, perinatal, or neonatal death review interventions (**Figure 1**). Humanitarian settings were defined as locations described as such by authors or contexts with a United Nations (UN) humanitarian appeal and/or refugee response efforts at the time evidence was collected [36].

We searched Scopus, Embase, and MEDLINE in December 2023for peer-reviewed literature that reported on the implementation of MPDSR and related death review interventions within humanitarian settings, including qualitative, quantitative, and mixed methods studies, reviews, field reports, and other relevant documentation of descriptive data. The search strategy incorporated two primary concepts: MPDSR and related death review interventions, and humanitarian settings (Appendix S1 in the **Online Supplementary Document**). We sourced grey literature from humanitarian organisations, governmental entities, UN agencies, and humanitarian web-portals (e.g. ReliefWeb and humanitarianresponse.info). We also screened the reference lists of relevant articles and queried subject matter experts within the humanitarian field for literature referrals. We restricted the literature search to English- and French-language documents published or produced from 1 January 2016 to 31 December 2023 to generate a synthesis of evidence since dissemination of WHO’s seminal report on global implementation of maternal death surveillance and response (MDSR) [37]. However, we did not exclude literature discussing implementation prior to 2016.

We then imported our search results into Covidence [38] and removed any duplicate records. Two independent researchers screened the title and abstract, followed by the full text of the studies against eligibility criteria (**Table 1**). One investigator (MM) resolved any screening discrepancies and extracted data using a piloted template. We did not conduct any appraisal or risk of bias assessment due to the descriptive focus of the scoping review.

**Table 1:** Eligibility criteria

Concept	Include	Exclude
Intervention	Literature reporting on or describing MPDSR and related death review interventions (**Figure 1**).	Literature reporting data from mortality surveys, mortality measurement or estimation reports, and cause of death determination methods and reports outside the context of routine surveillance.*
Context	Humanitarian contexts as described by authors; contexts with a humanitarian appeal and/or refugee response efforts at the time evidence was collected.	Settings without a humanitarian appeal or refugee response at the time of implementation; subnational contexts without a humanitarian or refugee response.
Timing	Published in 2016–23.	Published outside of the 2016–23 window.
Language	Published in English or French.	Published in other languages.

MPDSR – maternal and perinatal death surveillance and response

*For the purposes of this study, we defined routine surveillance as ‘the ongoing, systematic collection, analysis, and interpretation of health-related data essential to planning, implementation, and evaluation of public health practice’ [39].

We synthesised the literature using thematic tables constructed by country or humanitarian context to summarise key characteristics of each identified intervention, including intervention type; policy environment; governance or partnerships, implementation level; and phase (categorised as planning: pre-implementation; early to mid: 1–5 years of implementation; mid to late: >5 years of implementation, and pilot: (if indicated by the author)). We also outlined the implementation processes for each intervention by facility-based (the identification, reporting, and/or review of maternal and perinatal deaths that occurred within a health facility) and community-based (the notification, reporting, and/or review of maternal and perinatal deaths by community actors) approaches. Processes were delineated by key steps in the MPDSR cycle: identification, reporting, review, and response.

We likewise synthesised the data by implementation outcomes based on Proctor’s Model for Implementation Framework [40], which allows for analysis of how MPDSR interventions were intended to be implemented (adoption); the adherence to established MPDSR implementation plans (fidelity); the scale and integration of MPDSR interventions within health systems (penetration); how MPDSR interventions are institutionalised (sustainability); and the perceived fit or relevance of MPDSR interventions in humanitarian contexts (appropriateness) (**Table 2**). We identified factors influencing implementation by examining commonalities and differences in implementation experiences and assessing convergence or divergence with pre-determined programmatic assumptions [41].

**Table 2:** Key study outcome measures and constructs

Implementation outcomes	Definitions	Constructs	Salience by setting
Adoption	The uptake of MPDSR and related death review interventions from an organisational or implementation perspective.	Governance; policy adoption; facility-based implementation processes: identification, report, review, response; community-based implementation processes: notification, report, verbal and/or social autopsy, response; data systems and tools.	Any context.
Fidelity	The degree to which MPDSR and related death review interventions were implemented as intended, according to local, national, or international guidelines or action plans.	Adherence to MPDSR cycle or implementation processes; quality of reporting and response; implementing actor responsiveness.	Contexts in early-mid (1–5 years) or mid-late (>5 years) implementation phases.
Penetration	The integration of MPDSR and related death review interventions within health systems in humanitarian settings.	Positionality within health systems; interoperability with other systems (e.g. surveillance and health information systems).	Contexts in early-mid (1–5 years) or mid-late (>5 years) implementation phases.
Sustainability	The extent to which MPDSR and related death review interventions are institutionalised within a health system or humanitarian programming.	Local ownership of MPDSR and related death review interventions; sustained funding streams; institutionalised capacity.	Contexts in mid-late (>5 years) implementation phase.
Appropriateness	Perceived fit or relevance of MPDSR and related death review interventions within humanitarian settings.	Perceived relevance of MPDSR and related death review interventions within humanitarian contexts; perceived complexity of MPDSR and related death review interventions.	Contexts in planning or early-mid (1–5 years) implementation phases.

MPDSR – maternal and perinatal death surveillance and response

## RESULTS

**Figure 2:**
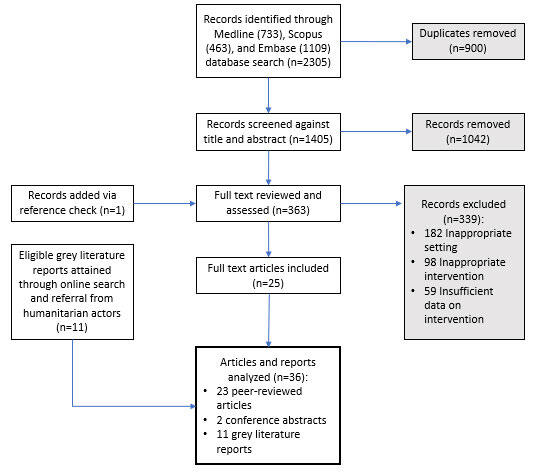
Search strategy flow diagram.

Our initial search retrieved 2305 records. After removing 900 duplicates, we screened the titles and abstracts of 1405 records and reviewed 364 in full, of which 25 met eligibility criteria [14–22,24–27,42–53] (**Figure 2**). Additionally, 11 grey literature reports identified via online search and referral from humanitarian experts met our inclusion criteria [54–64]. Thus, we abstracted and analysed data from 36 records in total, including 23 peer-reviewed articles, 2 conference abstracts, and 11 grey literature reports.

**Figure 3:**
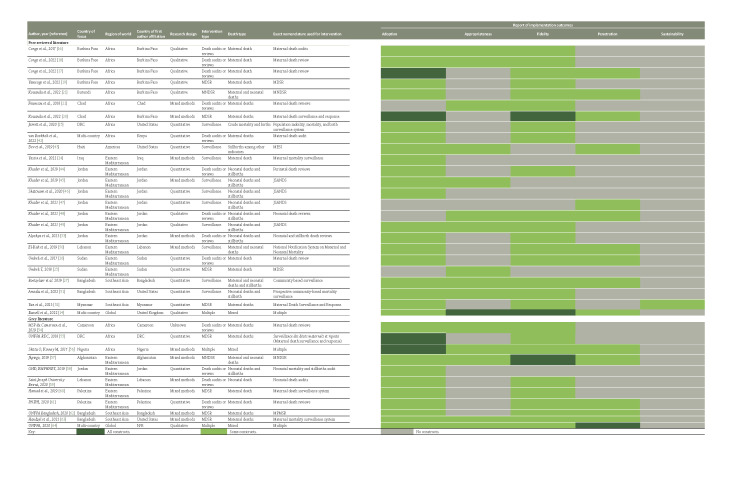
Key study outcome measures and constructs. DRC – Democratic Republic of Congo, EMPHNET – Eastern Mediterranean Public Health Network, GHD – Global Health and Development, JSANDS – Jordan stillbirth and neonatal death surveillance, MDSR – maternal death surveillance and response, MESI – integrated monitoring, evaluation, and surveillance system, MNDSR – maternal and neonatal death surveillance and response, MPMSR – maternal and perinatal mortality surveillance and response MSP – Ministere de la Sante Publique, N/R – not reported, PNIPH – Palestinian National Institute of Public Health, RDC – République Démocratique du Congo, UNFPA – United Nations Population Fund.

Approximately half (56%) of the literature described maternal, perinatal, and/or neonatal mortality surveillance and response interventions (MPDSR, maternal and neonatal death surveillance and response (MNDSR), MDSR, perinatal death surveillance response (PDSR), and surveillance); 36% described maternal, perinatal, and/or neonatal death review interventions (reviews, audits, or confidential inquiries); and 8% described multiple interventions. The majority of literature focussed on interventions only reporting and/or reviewing maternal deaths (47%). The eligible literature represented humanitarian contexts in Africa (33%), the Americas (3%), Eastern Mediterranean (44%), and Southeast Asia (14%), along with 6% of records describing interventions in humanitarian contexts globally. Among the peer-reviewed literature, 32% used qualitative methodology, 44% used quantitative methodology, and 24% used mixed methods approach. None of the identified articles/reports addressed all five implementation outcomes; documentation of constructs related to adoption and fidelity were best represented across the literature (**Figure 3**).

Across the 36 articles and reports, 33 unique programmes reported on 37 interventions within humanitarian contexts in 27 countries (Table S1 in the **Online Supplementary Document**). Five of the 33 programmes were reported in multiple articles/reports that either described multiple types of interventions and/or focussed on different pieces of an intervention (i.e. maternal deaths in one record and the full MDSR system in another), often using different nomenclature to specify the intervention (**Figure 3**). The majority of identified programmes (61%) only included the report and/or review of maternal deaths; less than (18%) focussed on stillbirths, neonatal, or perinatal deaths; while 21% combined foci to include maternal and neonatal death report/review [3] or maternal and perinatal deaths [4]. Overall, significant gaps were reported across all five implementation outcomes: adoption, appropriateness, fidelity, penetration, and sustainability.

### Adoption

The policy environment varied greatly among the 27 countries with reported interventions (Table S1 in the **Online Supplementary Document**). Policies, laws, and/or ministerial decrees related to the report or review of maternal and/or neonatal deaths were reported in records associated with half of the countries (n/N = 13/27). National or partner-specific implementation guidelines for MPDSR and related death review interventions were reported in 15 (56%) countries, while no information pertaining to the policy environment was found in articles/reports associated with identified programmes in four countries (Haiti, Lebanon, Myanmar, and Palestine).

Governance and partnerships of identified programmes implementing MPDSR and related death review interventions were also diverse, including actors from ministries of health, UN agencies, international non-governmental organisations, local organisations (e.g. research or academic institutions, consulting groups, and health professional societies), and humanitarian working groups. Ministries of health were reported to lead and/or support implementation of MPDSR and related death review interventions in 52% of programmes, while UN agencies, including the United Nations Population Fund (UNFPA) (30%), United Nations High Commissioner for Refugees (UNHCR) (18%), WHO (9%), and the United Nations International Children's Emergency Fund (UNICEF) (3%) were reported as leading agencies or partners in 39%.

The implementation phase and scope of identified programmes also varied: at the time of reporting, 3% were in the planning phase, 33% in the pilot phase, 36% in the early to mid (1–5 years) phase, 9% in the mid-scaled (≥6 years) phase, and 18% had no related information reported. Over a fifth (21%) of the programmes implemented MPDSR and related death review interventions at both facility and community levels, 67% were implemented at the facility level, and 12% were solely community-based interventions. Few programmes (n = 3) implemented a facility-based component at all levels of the health system (i.e. primary, secondary, and tertiary health facilities); that said, most programmes with facility-based interventions (52%) did not specify their health system level of implementation.

Overall, descriptions of the implementation processes associated with facility-based interventions varied widely in detail and yielded limited insights. The identification step, namely who is responsible and how deaths should be identified and notified to responsible actors within facilities, was described for over a third of the programmes (42%) (Table S2 in the **Online Supplementary Document**), while the processes related to reporting and reviewing of deaths were most frequently reported, in 58% and 76%, respectively. In particular, the establishment of review committees was documented for 84% of programmes: 52% had national review committees, 64% had subnational review committees (e.g. district or state levels), and 32% had facility-based review committees. Only 40% of identified programmes reported response mechanisms.

Community-based interventions were reported in 12 programmes across eight countries (Table S3 in the **Online Supplementary Document**). Yet, the implementation processes were specified in only half (n = 6) of the programmes, of which five were fully described (i.e. each relevant process documented in the literature). These programmes included the population mobility, mortality, and birth surveillance system in Democratic Republic of Congo (DRC) [15]; MNDSR in Afghanistan [57]; and two surveillance systems [27,51] and MDSR [62,64] in Cox’s Bazar (CXB), Bangladesh. Less than half (42%) of programmes described community-based death identification processes, most of which (n/N = 4/5) involved household visits by community health workers (CHWs), who were often then tasked with reporting deaths to their supervisor for verification and/or input into a surveillance database. Processes related to the conduct of verbal autopsies were only described for three programmes [27,57,62,64] which consisted of a CHW accompanying one or more skilled birth attendants (usually a midwife) to the household to conduct the verbal autopsy. Response processes were also only described for three interventions, all with different approaches to discussing, sharing, and addressing identified issues.

In addition to implementation processes, we investigated the availability and development of data systems or tools to understand the adoption of MPDSR and related death review interventions (Table S4 in the **Online Supplementary Document**). Many programmes reported on the use of tools adapted from international guidance [14,18,26,49,50,55,56,61], while identified MPDSR interventions led by UNHCR employed their own tools for reporting and review of maternal and perinatal deaths [42,64]. The unavailability of some tools was also reported in Burkina Faso [17,19], with a lack of tools to report and document neonatal death cases being a particular concern [19].

The use of systems for reporting deaths varied, with parallel or dual reporting systems described in contexts with multiple stakeholders (e.g. UNHCR and government MPDSR systems) [14,64]. Electronic or digital systems were used in some contexts – for example, the electronic Jordan stillbirth and neonatal mortality surveillance (JSANDS) system in Jordan was used for the reporting of stillbirths and neonatal deaths [44–49], while electronic dashboards were developed in Burundi to track maternal and perinatal deaths [21]. The use of systems for monitoring and evaluating response efforts was not reported in any context; in fact, a lack thereof was noted in relation to the MNDSR in Burundi [21], MDSR in Chad [20], and generally across humanitarian contexts by Russell et al. [14].

### Fidelity

Adherence to established implementation processes was low across many programmes (Table S4 in the Online Supplementary Document). Underreporting of maternal and perinatal deaths was reported by 39% of programmes [15,19–21,26,27,42,50–52,55,57,60] and summarised across programmes by Russell et al. [14] and the UNFPA [64], including the underreporting of deaths that occurred in transit or upon arrival at a higher-level facility after being referred for care [14]. Only a sample of reported maternal and perinatal deaths were reviewed in 33% of programmes [14,17–22,42,54–57,64]; however, facility-based reviews and community-based verbal autopsies were stated to have been conducted on all reported maternal deaths in the MDSR programme in CXB [62]. Delays in the notification or reporting of deaths [15,19,21,42,52,63] and in reviewing death cases [16,17,21,42,56,63] were documented in 18% of programmes and discussed as an issue across programmes reported by the UNFPA [64]. Limited uptake of recommendations and/or response efforts to address identified issues was also frequently reported [14,17,18,20–22,55,57,61,64]. In particular, two programmes (MDSR in DRC and maternal death reviews in Cameroon) documented weak transmission of information, recommendations, and proposed response from facilities to national level stakeholders [22,55].

Programme adherence was exacerbated by the poor quality of available data on maternal and perinatal death cases across contexts. Inadequate patient information in facility records was documented for 27% of programmes [17,18,21,26,42,44,56,57,59,61] and discussed as a general issue by Russell et al. [14] and UNFPA [64]. In particular, inaccessible information at referral facilities or health systems was reported as a data quality issue for three programmes: MPDSR in Burkina Faso, maternal death reviews in Palestine, and UNHCR death reviews across Africa [17,42,61]. Programmes also reported on missing or incomplete reporting on death review forms and tools [18,20,42,52,56,57,63] and misclassification of deaths [14,26,51,52,57,60,63,64]. Variation in stillbirth reporting was also highlighted by Russell et al. [14] as an issue across identified humanitarian contexts. Furthermore, the utilisation of consistent definitions of households was highlighted as a data quality issue for the community-based surveillance system in DRC [15], while the lack of reliable denominators to calculate mortality rates was attributed to population movement and distorted household sizes by community-based programmes in DRC and Bangladesh [15,27].

A lack of harmonisation of systems, interventions, and/or data sources related to maternal and perinatal death, especially between partners or with government systems, was reported to impact the quality of reporting and review of deaths in Burkina Faso, Uganda, and Palestine [19,61,64]. In Jordan and Palestine, local adaptations of international tools for reporting and reviewing maternal and perinatal deaths over-simplified data, creating significant gaps in data that was otherwise needed to adequately understand the death cases [49,61]; Russell et al. [14] also generalised this phenomenon across community-based approaches in regard to the simplification of verbal autopsy tools and suggested that the use of paper tools could facilitate the manipulation and/or concealment of data related to death cases. The unavailability of tools for maternal death reviews in Burkina Faso [17] and health information systems in Jordan [44] also created difficulties in maintaining fidelity to MPDSR and related death review interventions. In contrast, the MNDSR programme in Burundi leveraged dashboards to monitor mortality trends, yet no analysis or information related to death reviews were tracked using this tool [21]. The lack of disaggregation of data by setting (humanitarian vs development) and by population (refugee, internally displaced person, host community) was also identified in Sudan and Uganda as a data aresue limiting insights and preventing actors from tailoring response priorities based on the affected population [64]. Similarly, generic or broad recommendations resulting from death reviews often thwarted impactful response efforts in Burkina Faso [18] and across contexts reported by Russell et al. [14].

Limited health provider participation in MPDSR and related death review interventions was reported due to reluctance and various demotivating factors in 18% of identified programmes [17,19,20,42,44,49,57] and generalised across the Russell et al. [14] and UNFPA programme appraisals [64], including a lack of buy-in to the value of MPDSR interventions [17,44,57], a lack of or inadequacy in the uptake of recommendations and action points identified during reviews [14,17,57], a lack of financial support for health providers to participate [17,49,57,64], limited or lack of time to participate [14,44,57], the non-involvement of key staff in review and response activities [17], and concerns about potential scrutiny and damage of reputation [14,19].

In particular, limited participation in death review meetings was reported in Burkina Faso, Burundi, Chad, Jordan, Afghanistan, and across UNFPA programmes [16,17,20,21,49,57,64], often due to a lack of financial support for participants in the review sessions [17,49,57,64], lack of interest in participation by non-clinician actors [16], the silencing of voices of participating members during facility review meetings due to hierarchies [16], and a lack of supportive policies for the functionality of review committees [20,64]. Nonetheless, the JSANDS programme in Jordan reported active facility committee participation, yet emphasised a need for national review committees [48]. Moreover, incentivised MPDSR interventions employing performance-based financing for death reporting and reviews were reported to be successful in ensuring adoption and fidelity in Burundi and Afghanistan [21,54].

Discussion of community actor responsiveness and involvement was limited. Active participation of community actors (e.g. CHWs, surveillance agents, etc.) was reported in community-based surveillance and MDSR approaches in DRC and Bangladesh [15,27,64], with recognition that the use of local community actors facilitated community acceptability and improved the accuracy of data. Reported community involvement in facility-based approaches varied. For example, Van Boekholt et al. [42] reported that family members frequently (>50% of cases) participated in UNHCR facility maternal death audits. Meanwhile, limited to no community engagement in facility-based MPDSR and related death reviews, including a lack of community awareness of these systems, was reported to hinder the uptake of review and response steps in Chad and Afghanistan [20,57].

### Penetration

Overall, the institutionalisation of MPDSR interventions into health systems varied greatly across humanitarian settings [14,17,26,48,52,56,57,64]. Only in Uganda were the MPDSR interventions implemented by humanitarian partners fully integrated into the national government-led MPDSR system [64]. In some contexts, measures have been put in place to better integrate MPDSR interventions within the health system, including the integration of maternal and near-miss reviews in Palestine [64], making hospital accreditation dependent upon the establishment of neonatal death review committees in Jordan [48], institutionalisation of review committees within the health system in Cameroon [54], and linking MPDSR with quality improvement committees or activities in Nigeria and Afghanistan [56,57]. Yet collaboration and coordination across different levels of the health system (i.e. between review committees at facility, subnational, and national levels or between referring facilities) was limited across many settings [17,64].

Many interventions leveraged existing disease surveillance systems for the identification and reporting of maternal and perinatal deaths, including the integrated disease surveillance and response system [20,21,55]; WHO’s early warning, alert, and response system in CXB [27,62–64]; and the integrated monitoring, evaluation, and surveillance system in Haiti [43]. In addition, the maternal and child health e-registry, a comprehensive health information system for the national Ministry of Health primary clinics, was used in Palestine [61]. The civil registration and vital statistics system was employed to triangulate reported deaths in Jordan [47]; however, it was used to a lesser extent in other humanitarian contexts due to reported gaps or deficiencies in the registration of deaths [50,64].

### Sustainability

For this study, we defined sustainability constructs as the local ownership, sustained funding streams, and/or institutionalised capacity of interventions, and determined them to be relevant for examination only in the mid-late implementation phase (>5 years). Thus, the reported interventions applicable for analysis were the UNHCR maternal death audits conducted in refugee camps in sub-Saharan Africa [14,42], maternal and neonatal mortality surveillance of UNHCR hospitals in Lebanon [50], and MDSR and associated death reviews in Palestine (**Table 2**) [60,61,64]. Local ownership of the MPDSR and related death review interventions was reported in Palestine (Table S4 in the **Online Supplementary Document**), with the establishment and implementation of MDSR by the Ministry of Health [61]. While Van Boekholt et al. [42] noted that UNHCR encourages maternal death reporting and review in refugee contexts ‘in line with national approaches and in conjunction with ministries of health and partners,’ no further documentation of possible local ownership was discussed in relation to their programming. Similarly, there was no report of sustained funding streams or institutionalised capacity in the literature; instead, challenges related to shortages of financial and human resources were highlighted.

### Appropriateness

Over a third of programmes (36%) described the relevance of MPDSR and related death review interventions (Table S4 in the **Online Supplementary Document**), with most affirming relevancy in relation to the ability to understand health system performance, identify gaps in service delivery, and mount quality improvement activities to prevent future deaths [16,22,27,43,51,56,57]. Stakeholders from programmes in Jordan and Sudan also stressed MPDSR interventions’ potential to create change in policy and provider practice stemming from the learnings and findings from death reviews [25,48,49]. Authors also discussed the value of acquiring data on maternal and perinatal mortality within crisis-affected areas, emphasising opportunities to understand the magnitude of the crisis, identify marginalised populations, and address disparities in care, especially for refugees and internally displaced persons in Jordan, Uganda, and Sudan [45,49,64]. The review by Russel et al. [14] highlighted the relevancy of community-based interventions in understanding and addressing community and cultural factors influencing health decision-making. In addition, Boetzelaer et al. [27] underscored the importance of improving engagement to build trust between communities and humanitarian actors in Bangladesh. In contrast, the irrelevancy of MPDSR in humanitarian settings was discussed by stakeholders from the UNFPA Bangladesh country office [64], suggesting MPDSR is not considered a component of humanitarian duty but rather an intervention for development contexts.

Appropriateness was also discussed in terms of intervention complexity. The complexity of various forms and tools for use in these settings, where implementation often relies on lower-level health cadres, was acknowledged in Burkina Faso and across contexts by Russell et al. [14,18]. Also, the complexity of community-based approaches due to high personnel requirements [27] and hierarchical reporting and death verification systems [63] was reported in CXB.

### Factors influencing implementation outcomes

Across contexts, financial and human resources were documented as foundational drivers of implementation complexities. Insufficient funding was a challenge [14,16,17,20–22,55,60,64], and many programmes were plagued with short competitive funding cycles, with little stakeholder will to mobilise resources for MPDSR and related death review interventions amidst other overwhelming and often unmet service delivery needs [16,17,26,47]. In particular, the MDSR programme in Chad no longer functioned after the withdrawal of UNFPA financial support [20], while programming interruption was also projected in Palestine pending United Nations Relief and Works Agency (UNRWA) funding cuts [60]. Staffing shortages with high attrition rates, well-known in humanitarian contexts, also trickled down to impact MPDSR implementation across the majority of programmes [14,16,17,20,21,26,44,52,56,64]; combined with funding shortages, they resulted in low staff capacity and/or insufficient training [14,17–19,21,27,44,55,57,64]. High human resource and supervision needs were also identified by programmes implementing community-based approaches [15,27].

Leadership engagement at national, sub-national, and facility levels was identified as a key factor that influenced adoption and fidelity of MPDSR interventions in 19% of programmes and generalised across contexts reported by Russell et al. [14,19–21,26,27,57]. For example, actors implementing the MNDSR programme in Afghanistan attribute the attention, recognition, and support of the Ministry of Public Health to the programme’s success [57]. On the contrary, unstable political environments and lack of political will to mobilise funding hindered implementation of MPDSR interventions in Burundi [21]. In Yemen, the policy environment, deficient of legal protections for health providers partaking in MPDSR, was reported to deter implementation of MPDSR interventions and obstruct accountability, thwarting buy-in and engagement at many levels [64].

The synthesis by Russell et al. [14] suggests that facility administration buy-in for MPDSR programming was critical [14]. Yet some programmes reported limited administrative supervision, monitoring, and evaluation of interventions [17–21]. In Nigeria, this was described as supervisor ‘inertia to monitor maternal death review activities’ [56]. In Afghanistan, low administrative support translated into the promotion of MPDSR interventions as a punitive mechanism [54]. In Chad, demonstrated political will to support implementation of MDSR was obstructed by the lack of MDSR supervision at national, regional, and district levels [20]. Divergent priorities between partners and humanitarian actors implementing MPDSR interventions were also reported in Bangladesh, in addition to limited accountability at all levels of the systems, especially between humanitarian actors, in Chad, Sudan, and across the six UNFPA country programmes [20,26,61].

Leadership engagement and implementing actor responsiveness were reported to be highly dependent upon the pervasiveness of a blame culture. In Burkina Faso [16,19], Chad [20], Nigeria [56], and Afghanistan [57], but also generalised across contexts by Russell et al. [14] and UNFPA [64], actors at every level were concerned with blame and the resulting consequences related to reporting mortality, such as loss of funding or employment and/or fines, sanctions or other legal implications. Issues ensuring health provider confidentiality in Jordan [44] and anonymity in Burkina Faso [16,17] also influenced participation and the quality of maternal and perinatal death reviews. Hierarchies within death review committees exacerbated blame culture by fostering opportunities to pit rival clinicians against each other in defence of their provision of care in Nigeria [56], or reinforcing secrecy and misreporting when providers do not feel comfortable speaking freely while among superiors in Chad [20]. Falsification or omission of information related to death cases was a product of rampant blame culture across many contexts [14,19,57]. Health providers in Burkina Faso reported that death review meetings became highly stressful debates and arguments [16]; Russell et al. [14] also suggested that they caused psychological and moral distress. According to Russell and colleagues’ [14] cross-context synthesis and stakeholders in Afghanistan [57], leadership support of MPDSR interventions, and assurance of confidentiality were crucial in minimising blame culture.

Relationships between communities, health systems, and humanitarian actors also affected implementation outcomes. In Bangladesh [27,51], DRC [15], and generalised across contexts by Russell et al. [14], community-level deaths were often underreported or misreported due to mistrust in health systems and/or out of fear that their food rations and humanitarian aid may be affected by reductions in household size [14,15,27,51]. Socio-cultural norms and stigma associated with the acknowledgement and declaration of deaths (e.g. abortion-related maternal mortality, stillbirths, etc.) also caused underreporting of community-based deaths in Afghanistan and Bangladesh [51,57] along with fatigue from ongoing household visits by multiple actors over long surveillance periods in Bangladesh and DRC [15,27]. Russell et al. [14] emphasised that, when a maternal death occurs in contexts with socio-ethnic conflict, tensions between communities and health providers of opposing ethnic groups may amplify insecurity, ethnic fighting, tribal wars, and/or result in retaliation against facilities and health providers.

## DISCUSSION

This scoping review shows a vast landscape of MPDSR and related death review interventions within humanitarian settings in 27 countries representing 69% of countries with a 2023 UN humanitarian appeal [36]. Mixed and/or evolving nomenclature and programming within the same context mimics the global shift from maternal death reviews or mortality surveillance to more comprehensive MPDSR interventions incorporating report, review, and response to maternal and perinatal deaths with a quality improvement perspective [1–4]. However, similar to the larger body of evidence on implementation in LMICs [28,30,32], the adoption of this comprehensive approach is still young; due to difficulties in the identification and review of perinatal deaths, only 9% of programmes have adopted the full MPDSR system, with some (6%) attenuating challenges by replacing the ‘P’ for an ‘N’ (MNDSR). Nonetheless, with the publication of WHO guidelines on the audit and review of stillbirths and neonatal deaths in 2016 [4], an emergence of a cluster of programmes (15%) focussed only on stillbirths, neonatal, or perinatal deaths was found shortly afterwards.

Overall, the literature base suffers from limited and inconsistent reporting on the implementation of MPDSR and related death review interventions, as documented in other reviews [28,32]. In particular, the lack of documentation on adoption (namely implementation processes) hinders synthesis and limits learnings on how MPDSR and related death review interventions were designed and adapted fit-for-context across the diverse humanitarian settings. Along with gaps in reporting on adoption, many fidelity and penetration constructs reported by Russell et al. [14] and UNFPA [64] were generalised across 66% of identified programmes, limiting insights gleaned from linkages between context-specific MPDSR policy environments, governance and partnerships, implementation processes and programmatic adherence and integration.

Underlying obstacles of limited funding, thoroughly documented in LMICs [28,31,32], are exacerbated within humanitarian contexts by short funding cycles, competitive priorities, and complex funder dynamics. Overburdened staff within high attrition contexts was also systematically reported in the literature [28,30–32]; however, the comparative magnitude of the staffing crisis faced in humanitarian settings is not discernible. Our findings also reflect the comprehensive evidence base on blame culture [28–32]; in reported programmes across humanitarian contexts, health providers and review committee members could be demotivated not only by their leadership but also by unsupportive implementation environments rife with blame culture and unrealistic workloads, given existing resources or the lack thereof [14,17,19,20,42,49,57,64].

However, amidst the backdrop of extreme resource shortages and pervasive blame culture in humanitarian settings, promising practices in adapting and tailoring MPDSR programming fit-for-context are observable within the literature. For example, humanitarian inter-agency groups (e.g. sexual and reproductive health working group) were leveraged in CXB for technical support and implementation accountability in the absence of a national MPDSR programming or response systems [61]. Adaptations have also been made to account for lower-level and/or lower-literacy health cadres (e.g. community health workers and volunteers) often implementing these interventions in humanitarian contexts [14,49,61]; however, Russell et al. [14] question whether these pragmatic adaptations were made at the expense of evidence-based high-quality MPDSR programming. In addition, the design of the MDSR programme in CXB, with complete integration of WHO’s early warning, alert, and response system, has not only centralised MDSR data collection across the numerous camps and implementing partners, but allowed for triangulation between community- and facility-based reporting, ultimately identifying many unreported facility-based deaths [27,62–64]. Given the reported human resource shortages in humanitarian settings, some programmes also created smaller death review committees with rotating membership, so as to reduce the burden of participation [14,16,54]. Overall, the shift in framing MPDSR and related death review interventions as a quality improvement approach (instead of mortality estimation mechanism) [15,64] and/or programmatic integration within quality improvement teams [56,57] allows for the flexibility of continuation, delay, and/or pause of the implementation during fluctuating levels of security or crisis, without detriment to prior reporting and reviews.

At the community level, dynamics between community members and humanitarian and health facility actors are convoluted; intertwined dependency on humanitarian aid and services alongside socio-cultural tensions and practices surrounding death often translates into mistrust, stigma, and underreporting of maternal and perinatal deaths within communities [14,15,27,51]. Checchi et al. [65] also identified the dynamics between community-based deaths and ration reductions within the mortality estimation literature; however, insights are limited regarding socio-cultural underpinnings for underreporting or misreporting of maternal and perinatal deaths.

An expert consultation of humanitarian actors held in 2019 to share MPDSR implementation experiences from crisis-affected settings confirmed the value of MPDSR and recommended that programming be pursued, albeit only in protracted settings [66]. Evidence documented in terms of the relevancy of MPDSR and related death review approaches in humanitarian settings from this scoping review supports this stance [16,22,25,27,43,45,48,49,51,56,57,64], especially in regard to identifying marginalised populations and addressing disparities in maternity care for refugees and internally displaced persons [45,49,64]. Nonetheless, some humanitarian actors still debate the value add and appropriateness of MPDSR when their mandate is limited to life-saving health service provision [64].

This scoping review is not without limitations. Some relevant evidence may have been missed due to the ever-expanding nomenclature and terminologies used to describe MPDSR and related death review interventions. Limiting our literature search to publications in 2016–22 may have excluded implementation evidence on earlier renditions of MPDSR (e.g. maternal death reviews, audits, and confidential inquiries) that would still be applicable to more comprehensive systems promoted in WHO’s most recent report on global implementation of MDSR [37]. Similarly, identified MPDSR and related death review interventions were only found in one of six Latin America countries with a 2023 UN appeal, which could be due to search limitations that excluded literature in Spanish (and other languages outside of English and French).

## CONCLUSIONS

While a substantial programmatic landscape of MPDSR and related death review interventions was identified within humanitarian settings in 27 countries, extensive gaps in evidence exist pertaining to adoption, fidelity, penetration and sustainability of these interventions. Emergent implementation dynamics unveil a complex interplay between humanitarian actors, communities, and the health system, worthy of in-depth investigation. Future mixed methods research evaluating the gamut of identified MPDSR programmes in humanitarian contexts will greatly bolster the evidence base. Investment in comparative health systems research to understand how best to adapt MPDSR and related death review interventions to crisis affected contexts is a crucial next step.

## Additional material


Online Supplementary Document
