# Supplementary material for: Implementation of maternal and perinatal death surveillance and response and related death review interventions in humanitarian settings: A scoping review
Source: J Glob Health. 2024 Jul 12;14:04133. doi: 10.7189/jogh.14.04133 (PMC11239189; doi:10.7189/jogh.14.04133)
Supplement: Online Supplementary Document [file jogh-14-04133-s001.pdf]

SUPPLEMENTARY DOCUMENT

Table S1: Landscape of reported MPDSR and related death review interventions (ordered by region and country)

Reported interventions by program	Implementation location within humanitarian contexts	Reported MPDSR policies & guidelines	Reported intervention governance/ partners	Implementation level	Implementation phase (years)	Report of implementation outcomes[a]				
						Adoption	Appropriate-ness	Fidelity	Penetra-tion	Sustain-ability
Africa										
Burkina Faso										
Maternal death reviews (16–18) & MDSR (19)	Dafra, Djibo, Ouahigouya, Tenkodogo, and Tougan	National standards for clinical audit (2010); National MDSR guidelines (year N/R)	Ministry of Health	Facility (primary, secondary and tertiary level)	Early-mid (2010-2015)					N/A
Burundi										
MNDSR (21)	Bujumbura and Gitega	National MNDSR guidelines and Ministerial decree to establish national MNDSR committee (2015)	Ministry of Health under National Reproductive Health Program	Facility (secondary and tertiary level)	Early-mid (Pilot) (2015-2017)					N/A
Cameroon										
Maternal death reviews (14,53)	National	National policy/law on maternal death, neonatal death, and stillbirth reviews (year N/R)	Ministry of Public Health	Facility (primary, secondary, and tertiary level)	Early-mid (2014-2017)					N/A
Chad										
Maternal death reviews (14,22) & MDSR (20)	Moundou, Mongo, N'Djamena, and Koumra regions	National policy/law on maternal death, neonatal death, and stillbirth reviews (year N/R) Ministerial order obliging notification and audits of maternal and neonatal	Ministry of Public Health with UNFPA support	Facility (tertiary level)	Early-mid (Pilot) (2012-2016)					N/A

Reported interventions by program	Implementation location within humanitarian contexts	Reported MPDSR policies & guidelines	Reported intervention governance/ partners	Implementation level	Implementation phase (years)	Report of implementation outcomes[a]				
						Adoption	Appropriate-ness	Fidelity	Penetra-tion	Sustain-ability
		deaths (2015); Ministerial order creating technical committee (2016)								
Central African Republic										
Community-based mortality surveillance of abortion-related maternal deaths (14)	N/R	N/R	N/R	Community	N/R					N/A
MDSR (63)	National	National policy to notify and review maternal deaths (year N/R)	Ministry of Health with UNFPA support	Facility	N/R					N/A
Democratic Republic of Congo (DRC)										
MDSR (14,54)	National	National MDSR Guidelines (year N/R)	Ministry of Health with UNFPA, WHO, and other donor support	Facility and community	Early-mid (2015-2018)					N/A
Population mobility, mortality, and birth surveillance system (15)	Fizi Health Zone, South Kivu	N/R	N/R	Community	Early-mid (Pilot) (2011-2012)					N/A
Mozambique										
MPDSR[b] (14)	N/R (Post-cyclone)	National policy/law on maternal and neonatal death reviews (year N/R)	N/R	Facility	N/R					N/A
Nigeria										
Maternal death reviews (PRRINN-MNCH) (55)	Yobe	Maternal death reviews (MDR) policy and guidelines (2013); MPDSR policy and guidelines (2016)	Save the Children and GRID Consulting	Facility (secondary and tertiary level)	Early-mid (Pilot) (2010-2013)					N/A
Maternal death reviews (MNCH2) (55)	Yobe		State Ministries of Health	Facility and community	Early-mid (2014-2016)					N/A

Reported interventions by program	Implementation location within humanitarian contexts	Reported MPDSR policies & guidelines	Reported intervention governance/ partners	Implementation level	Implementation phase (years)	Report of implementation outcomes[a]				
						Adoption	Appropriate-ness	Fidelity	Penetra-tion	Sustain-ability
MPDSR (14,55)	Yobe, Adamawa and Borno		Federal Ministry of Health and State Ministries of Health	Facility	Planning (2016)					N/A
Maternal death reviews (MSS) (55)	Yobe, Adamawa, and Borno		National Primary Health Care Development Agency	Facility (primary level)	Early-mid (Pilot) (2011-2013)					N/A
Sierra Leone										
MPDSR (14)	N/R (Ebola/post-Ebola)	National policy/law on maternal death reviews (year N/R)	N/R	Facility	Early-mid (2014-2019)					N/A
South Sudan										
MDSR (14,63)[b]	N/R	National policy to review maternal deaths (year N/R)	Humanitarian SRH working group	Facility and community	Early-mid (2013-2018)					N/A
Uganda										
MDSR (63)	Refugee settlements	National MPDSR guidelines (2017)	Ministry of Health, UNHCR, UNFPA	Facility	N/R					N/A
Multi-country										
Maternal death audits (14,41)	Refugee camps in: Burundi, Cameroon, Chad, Djibouti, Ethiopia, Kenya, Niger, Rwanda, South Sudan, Tanzania, and Uganda	Maternal death audit guidance (2007)	UNHCR	Facility and community [c]	Mid-late (2007-2019)		N/A			
Americas										
Haiti										
Surveillance of stillbirths (among other indicators) (42)	Sud, Nippes, Grand Anse departments	N/R	Ministry of Public Health and Population	Facility (secondary and tertiary level)	N/R					N/A
Eastern Mediterranean										
Afghanistan										

Reported interventions by program	Implementation location within humanitarian contexts	Reported MPDSR policies & guidelines	Reported intervention governance/ partners	Implementation level	Implementation phase (years)	Report of implementation outcomes[a]				
						**Adoption**	**Appropriate-ness**	**Fidelity**	**Penetra-tion**	**Sustain-ability**
MNDSR (14,56)	Herat, Kandahar, and Bamyan Provinces	MNDSR Guidelines (2014)	Ministry of Public Health with support from HEMAYAT, UNICEF, WHO and UNFPA	Facility (secondary and tertiary level) and community	Early-mid (Pilot) (2018-2019)					N/A
Iraq										
Maternal mortality surveillance & confidential enquiry (14,24)	National	National policy/law on maternal and neonatal death and stillbirth reviews (year N/R)	Ministry of Health	Facility	N/R					N/A
PDSR (14)	"Stable districts"		"Nationally led"	Facility	Early-mid (Pilot) (2018-2019)					N/A
Jordan										
Neonatal death and stillbirth audits (14,57)	Zatari and Azraq Syrian refugee camps	National policy/law on maternal death reviews (year N/R)	UNHCR and CDC	Facility (secondary and tertiary level)	Early-mid (2016-2019)					N/A
Stillbirth and Neonatal Death Surveillance (43–46,48) & death reviews (47,52)	Mafraq governorate (and other northern governorates), including Syrian refugees	No policy on perinatal death review in place	Jordan University of Science & Technology and Ministry of Health	Facility (tertiary level)	Early-mid (Pilot) (2019 – 2020)					N/A
Lebanon										
Maternal and Neonatal Mortality Surveillance (49)	National, including Syrian refugees	N/R	Ministry of Public Health in collaboration with Lebanese Society of Obstetrics and Gynecology, syndicate of private hospitals, and UNFPA	Facility	Mid-late (2004-2018)		N/A			
Neonatal death audits (58)	Refugees in Beirut Mount Lebanon, Bekaa, South and North Lebanon	N/R	UNHCR	Facility (secondary and tertiary level)	Early-mid (Pilot) (2019-2020)					N/A

Reported interventions by program	Implementation location within humanitarian contexts	Reported MPDSR policies & guidelines	Reported intervention governance/ partners	Implementation level	Implementation phase (years)	Report of implementation outcomes[a]				
						**Adoption**	**Appropriate-ness**	**Fidelity**	**Penetra-tion**	**Sustain-ability**
Palestine										
MDSR (59,63) & maternal death reviews (60)	National	N/R	Ministry of Health with support from UNFPA	Facility (secondary and tertiary level with NICUs)	Mid-late (2009-2019)		N/A			
Sudan										
MDSR (25,26,63)	18 states in Sudan	National policy to notify and review maternal deaths (year N/R)	Ministry of Health with support from UNFPA	Facility and community	Early-mid (2014-2017)					N/A
Syria										
Maternal death review (14)	N/R	National policy/law on maternal and neonatal death reviews (year N/R)	N/R	Facility	Early-mid (2015-2017)					N/A
Yemen										
MDSR (63)	Hadhramaut	Maternal death review guidelines (year N/R)	UNFPA	Facility	Pilot (date N/R)					N/A
South-East Asia										
Bangladesh										
Community-based surveillance of maternal and neonatal deaths and stillbirths (27)	13 Rohingya refugee camps in CXB	N/R	Médecins sans Frontières	Community	Early-mid (2017-2019)					N/A
MDSR (14,61–63)	All Rohingya refugee camps in CXB	National policy to notify and review maternal deaths (year N/R)	UNFPA, UNHCR, WHO, CDC, CIPRB, technical working groups	Facility (primary, secondary, and tertiary level) and community	Early-mid (2018-2020)					N/A
Community-based surveillance of neonatal deaths	29 Rohingya refugee camps in CXB	N/R	CDC with support of UNFPA, WHO, UNHCR, Ministry of	Community	Early-mid (Pilot) (2017-2018)					N/A

Reported interventions by program	Implementation location within humanitarian contexts	Reported MPDSR policies & guidelines	Reported intervention governance/ partners	Implementation level	Implementation phase (years)	Report of implementation outcomes[a]				
						Adoption	Appropriate -ness	Fidelity	Penetra- tion	Sustain- ability
and stillbirths (50)			Health, and NGOs							
Myanmar										
MDSR (51)	National	N/R	Ministry of Health	Facility	Early-mid (2017-2019)					N/A
Key: All constructs 50-99% of constructs 1-49% of constructs No constructs										

Notes:
Abbreviations: CDC: Centers for Disease Control and Prevention; CIPRB: Center for Injury Prevention and Research Bangladesh; CXB: Cox's Bazar; DRC: Democratic Republic of Congo; HEMAYAT: Helping Mothers and Children Thrive Project; MDSR: Maternal Death Surveillance and Response; MNCH2: Nigerian Maternal, Neonatal, and Child Health programme; MNDSR: Maternal and Neonatal Death Surveillance and Response; MPDSR: Maternal and Perinatal Death Surveillance and Response; MSS: Midwifery Service Scheme; N/R: Not Reported; NGO: Non-governmental organizations; NICU: Neonatal Intensive Care Units; PDSR: Perinatal Death Surveillance and Response; PRRINN-MNCH: Partnership for Reviving Routine Immunisation in Northern Nigeria and Maternal Newborn and Child Health Initiative; UNFPA: United Nations Population Fund; UNHCR: United Nations High Commissioner for Refugees; UNICEF: United Nations Children's Fund; WHO: World Health Organization
[a] *Analysis of each implementation outcome was completed according to its salience for the identified implementation phase. Not applicable (N/A) is noted where analysis for the implementation outcome is not indicated. Constructs for adoption, appropriateness, fidelity, and penetration were analyzed for programs with no reported implementation phase.*
[b] *Discussed under umbrella of MPDSR, yet vague description of specific type of intervention*
[c] *All listed countries participated in facility-based approaches; it is unclear which countries employed community-based approaches; as such, this program was excluded from S3 Table.*

Table S2: Implementation processes of reported *facility-based* MPDSR and related death review interventions (ordered by region and country)[a]

Reported interventions by program	Identification of deaths	Report of deaths	Death review	Facility review committee	Subnational review committee	National review committee	Response	All relevant steps described [b]
Africa								
Burkina Faso								
Death reviews (16–18) & MDSR (19)	Head of the maternity unit notifies the head of the health facility (19)	Health facility reports to the district health office, who reports to the national level on weekly basis (19)	Data collected and synthesized before review (18); Biannual reviews (19) or facility audits every 1-3 months (16)	Yes (16,17)	Yes – district committees (18,19)	N/R	Share findings from death reviews with health facility staff to develop solutions (16)	No
Burundi								
MNDSR (21)	Deaths notified to the Health information system (HIS) officer through the Integrated Disease Surveillance and Response (IDSR) and forwarded to the HIS district officer within 24 hours.	HIS officers at the district level report to the provincial health offices who will transmit the information to the national health information system (NHIS). The NHIS reports to the national reproductive health program office on weekly basis.	Monthly facility reviews lasting up to 3 hours discussing 2 death cases per review	Yes	No	Yes	Recommendations and action plans developed at facility level with CHWs, health providers, and managers	Yes
Cameroon								
Maternal death reviews (14,53)	N/R	N/R	N/R	N/R	Yes (14)	Yes (14)	N/R	No
Chad								
Maternal death reviews (14,22) & MDSR (20)	Deaths notified through IDSR within 24-48 hours of their occurrence (20)	Heads of health facilities report deaths to focal points among health facility managers on a weekly basis. The focal points report to the district epidemiological surveillance focal point, who in turn reports to the regional head of the epidemiological surveillance branch. At	Data collected by epidemiological surveillance and reproductive health focal points; reviews carried out within 30 days of notification; death reports sent to national level (20)	Yes (20)	Yes (14)	Yes (14,20)	N/R	No

Reported interventions by program	Identification of deaths	Report of deaths	Death review	Facility review committee	Subnational review committee	National review committee	Response	All relevant steps described [b]
		the regional and national hospital levels, the epidemiological surveillance focal points send the data directly to regional head of the epidemiological surveillance branch(20)						
DRC								
MDSR (14,54)	N/R	N/R	National MDSR committee holds quarterly meetings with stakeholders, UNFPA, and WHO (54)	N/R	Yes – provincial committees (54)	Yes (54)	National reports published (54)	No
Mozambique								
MPDSR* (14)	N/R	N/R	N/R	N/R	Yes	Yes	N/R	No
Nigeria								
Maternal death reviews (PRRINN-MNCH) (55)	Health providers notify the facility quality improvement (QI) team chair of deaths	N/R	Facility QI team lead collects the information from the case file and conducts interviews, when necessary. QI teams review findings and develop recommendations. Cases are subsequently discussed at quarterly local government area (LGA) MDR meetings	Yes	Yes – LGA committees	No	Recommendations are communicated to facility and health system leadership to improve quality of care.	No
MPDSR (14,55)	N/R	N/R	N/R	N/R	Yes (14)	Yes (14)	N/R	No
Maternal death reviews (MSS) (55)	Facility MDR committee members are notified of deaths	Facility MDR Committees report deaths via Notification Forms	Facility MDR Committees conduct review on each death and develop recommendations.	Yes	Yes – state committees	Yes	Facility MDR Committees develop action plans from their reviews and ensure their implementation at both facility and community levels. State MDR Committees (4)	Yes

Reported interventions by program	Identification of deaths	Report of deaths	Death review	Facility review committee	Subnational review committee	National review committee	Response	All relevant steps described [b]
							support stakeholders in determining root causes of the deaths, and introduce preventive measures based on findings and recommendations. National debriefing meetings are also held to compile and disseminate findings and mobilize resources for response.	
Sierra Leone								
MPDSR (14)	N/R	N/R	N/R	N/R	Yes	Yes	N/R	No
South Sudan								
MDSR* (14,63)	N/R	Deaths reported to HMIS (63)	N/R	N/R	Yes (14)	N/R	N/R	No
Uganda								
MDSR (63)	N/R	Deaths reported to UNHCR public health officer and through HMIS	Confidentiality agreement signed before death reviews at facility	Yes	N/R	N/R	Review results are shared; response activities (e.g., supply procurement and training) are tailored to context to improve service provision	No
Multi-country								
Maternal death audits (14,41)	N/R	N/R	Death audit forms are completed and submitted to national authorities and UNHCR personnel responsible for each camp/settlement (41)	N/R	N/R	N/R	On a yearly basis, audit reports are compiled and examined. Recommendations are developed and shared with UNHCR country operations and partners (41)	No
Americas								
Haiti								
Surveillance of stillbirths (among other indicators) (42)	N/R	Data from maternity registers are aggregated and uploaded to MESI	N/A	N/A	N/A	N/A	N/A	No

Reported interventions by program	Identification of deaths	Report of deaths	Death review	Facility review committee	Subnational review committee	National review committee	Response	All relevant steps described [b]
		during routine reporting						
Eastern Mediterranean								
Afghanistan								
MNDSR (14,56)	Each site's MNDSR focal person notifies provincial authorities via SMS and completes a death notification form (56)	Staff complete a death review form using information from health records or registers and consultation with staff and family members within 7 days of the death (56)	Facility MNDSR committees meet within 1 week of reported death to review completed facility death review forms, identify causes of death, contributing factors and corrective actions or response measures. On a monthly basis, provincial MNDSR committees also review deaths (56).	Yes (56)	Yes – province committees (14,56)	Yes (56)	Provincial MNDSR committees monitor the implementation of health facility action plans and raise issues of concern at Provincial Health Coordination Committee meetings. The national MNDSR steering committee also develops technical guidance and operational plans for MNDSR implementation, prepares bi-annual reports, and organizes national seminars to address findings and recommendations for action (56)	Yes
Iraq								
Maternal mortality surveillance & confidential enquiry (14,24)	Each death should be notified by the obstetrician or birth attendant within 24 hours (24)	N/R	Directorate of Health committee reviews case within 1 days to evaluate the service provided, identify the areas of under care and to act accordingly. The report is sent to the Central Committee for further analysis (24).	No (24)	Yes – directorate committees (14,24)	Yes (14,24)	N/R	No
Jordan								
Neonatal death and stillbirth audits (14,57)	N/R	Deaths reported to EMPHNET (57)	An EMPHNET investigator and assistant conduct field visits within 72 hours of reported deaths to complete the audit form. Interviews with mothers or caregivers are conducted and medical	No	No	No	N/R	No

Reported interventions by program	Identification of deaths	Report of deaths	Death review	Facility review committee	Subnational review committee	National review committee	Response	All relevant steps described [b]
			files reviewed. The audit form is submitted electronically to UNHCR (57)					
Stillbirth and Neonatal Death Surveillance (43–46,48) & death reviews (47,52)	N/R	Birth attendants complete a form on the electronic system (JSANDS) and assign the cause of death (44,46)	Monthly death review meetings are held to discuss detailed summaries of death cases and to identify critical delays, modifiable factors, and recommendations and actions (45,47,52).	Yes (47,52)	No (47,52)	No (47,52)	Implementation and monitoring plans are developed to address identified actions and recommendations from death reviews (46,47,52)	No
Lebanon								
Maternal and Neonatal Mortality Surveillance (49)	N/R	Vital data observatory focal persons (staff in public and private hospitals) report deaths to the MOPH Primary Care Department on a monthly basis	The national committee assigns an expert from the Lebanese OBGYN Society to investigate the case (interviews with medical team and chart review) and prepare a medical and/or technical report. The reports are reviewed during committee meetings every 4–6 months to validate the cause of death and develop recommendations.	No	No	Yes	N/R	No
Neonatal death audits (58)	N/R	N/R	Field investigators conduct audits via phone interviews with the parents or relatives and review medical records. A medical consultant reviews the information and submits an audit form to a coordinator and UNHCR.	No	No	No	N/R	No
Palestine								
MDSR (59,63) & maternal death reviews (60)	Deaths of women of reproductive age (15-49) are	The MCH team conducts a visit to facility and completes	The technical committee classifies the maternal deaths using the ICD-10	No (60)	No (60)	Yes (60)	N/R	No

Reported interventions by program	Identification of deaths	Report of deaths	Death review	Facility review committee	Subnational review committee	National review committee	Response	All relevant steps described [b]
	notified to the MCH team (MCH doctor and MCH nurse supervisor) at the MoH Primary Health Care district office (60,63).	an investigation form, attaching all related medical reports and the death notification form (death registry). The MCH team sends the form to the regional PHC office for review by a technical committee at the MoH Community Health Department (60).	and sends the cases to the National Maternal Mortality Committee, who reclassifies the for review and analysis (59,60).					
Sudan								
MDSR (25,26,63)	Deaths were notified by designated focal points via phone (26)	Information on maternal deaths is collected using standardized forms and reported to state review committees (26)	State maternal death review committees review all notified deaths to generate recommendations for improvement and then submit reports to the national maternal death review committee (26).	No (26)	Yes – state committees (26)	Yes (26)	N/R	No
Syria								
Maternal death review (14)	N/R	N/R	N/R	N/R	Yes	N/R	N/R	No
Yemen								
MDSR (63)	N/R	N/R	Investigation of reported deaths by UNFPA SRH coordinator	N/R	N/R	N/R	N/R	No
South-East Asia								
Bangladesh								
MDSR (14,61,63)	Deaths are notified by health facilities via EWARS (61)	Maternal deaths in EWARS are reported to the MPMSR committee, a camp-wide multi-partner review committee (61)	MPMSR committee supervises data collection, review, and analysis to develop recommendations (61,63)	No (61)	Yes – camp-wide/multi-partner committee (61,63)	No (61)	Findings and recommendations from reviews disseminated via SRH-working group, who also serves as accountability mechanism to ensure appropriate response (63)	Yes
Myanmar								

Reported interventions by program	Identification of deaths	Report of deaths	Death review	Facility review committee	Subnational review committee	National review committee	Response	All relevant steps described [b]
MDSR (51)	Deaths are identified by the physician present at the time of death	Physician of the hospital within the area of the maternal death notifies the respective state or regional health department and the central MDSR team within 24 hours	The MDSR team at the township (sub-division of a district) level conducts a field investigation at the place of maternal death within 21 days of the maternal death and sends data to state and regional MDSR review committees.	No	Yes – state and regional levels	No	N/R	No

Notes:

Abbreviations: DRC: Democratic Republic of Congo; EMPHNET: Eastern Mediterranean Public Health Network; EWARS: Early Warning Alert and Response system; ICD-10: International Classification of Diseases, 10th revision; LGA: Local government area; MCH: Maternal and Child Health; MNCH2: Nigerian Maternal, Neonatal, and Child Health programme; MoH: Ministry of Health; MSS: Midwifery Service Scheme; N/R: Not Reported; PRRINN-MNCH: Partnership for Reviving Routine Immunisation in Northern Nigeria and Maternal Newborn and Child Health Initiative

[a] *Only programs with reported steps of the implementation process are detailed in the table (20/28)*

[b] *Implementation processes were analyzed according to their relevancy to the identified intervention. For example, the review and response steps were not analyzed for surveillance systems responsible only for identification and report of maternal deaths. Not applicable (N/A) is indicated in these scenarios*

Table S3: Implementation processes of reported *community-based* MPDSR and related death review interventions (ordered by region and country) [a]

Reported interventions	Identification of death	Report of death	Review/VA of death	Response	All relevant processes described [b]
Africa					
DRC					
Population mobility, mortality, and birth surveillance system (15)	Households notified deaths during monthly visits by CHWs	Deaths documented in field books, verified by field supervisor, and reporting to surveillance database	N/A	N/A	Yes
Uganda					
MDSR (63)	N/R	Deaths are reported to a UNHCR public health officer and through the HMIS	N/R	N/R	No – partial description
Eastern Mediterranean					
Afghanistan					
MNDSR (14,56)	CHW are notified of deaths within the communities they serve (56)	CHWs report death via SMS to facility-based Community Health Supervisor (56)	The Community Health Supervisor and a skilled birth attendant visit the household of the deceased within 15 days of the notification to complete a verbal autopsy form (56)	Verbal autopsies are discussed during facility MNDSR committee meetings to develop recommendations and action plans. Provincial MNDSR committees monitor the implementation of health facility action plans and raise issues of concern at Provincial Health Coordination Committee meetings. The national MNDSR steering committee also develops technical guidance and operational plans for MNDSR implementation, prepares bi-annual reports, and organizes national seminars to address findings and recommendations for action (56)	Yes – by 1 record
South-East Asia					
Bangladesh					
Community-based surveillance of maternal and neonatal deaths and stillbirths (27)	Households notify surveillance workers of deaths during monthly (every 4 weeks) visits	Surveillance workers submit reports to surveillance team leaders who report cases to the Medical Response Team of midwives	Medical Response Team of 3 midwives conduct verbal autopsy	Findings published in biweekly MSF internal bulletin to facilitate targeted interventions and response.	Yes
MDSR (14,61–63)	Households notify CHWs of deaths during bi-weekly visits (63)	CHWs inform the CHW supervisor who verifies the information and reports cases to the reference health facility and the UNFPA MPMSR coordinator. (63); Report is also uploaded into EWARS (61)	A community midwife accompanied by the CHW conducts a verbal autopsy at the household 10 to 14 days after death notification. Findings reviewed by	Recommendations from reviews and verbal autopsies disseminated via SRH-working group, who also serves as accountability mechanism to ensure appropriate response (63)	Yes – across 2 records

Reported interventions	Identification of death	Report of death	Review/VA of death	Response	All relevant processes described [b]
			MPMSR sub-committee (61,63)		
Community-based surveillance of neonatal deaths and stillbirths (50)	Households notify CHWs of deaths during weekly visits	CHWs inform CHW supervisor who verifies the information and reports cases to the nearest health facility	N/A	N/A	Yes

Notes:
Abbreviations: EWARS: Early Warning Alert and Response system; MNCH2: Nigerian Maternal, Neonatal, and Child Health programme; N/R: Not Reported; VA: Verbal autopsy
[a] *Only programs with reported steps of the implementation process are detailed in the table (6/12)*
[b] *Implementation processes were analyzed according to their relevancy to the identified intervention. For example, the review and response steps were not analyzed for surveillance systems responsible only for identification and report of maternal deaths. Not applicable (N/A) is indicated in these scenarios*

Table S4: Synthesis of implementation domains addressed

Construct	# of articles or reports	Summary of findings
Adoption: *The uptake of MPDSR and related death review interventions from the organizational or implementer perspective*		
Governance	--	*S1 Table*
Policy adoption	--	*S1 Table*
Implementation processes	--	*S2 & S3 Tables*
Data systems and tools	*20*	*Tools:*<br>• *Tools adapted from international guidance* (14,18,26,48,49,54,55,60)<br>• *UNHCR tools used for reporting and review of maternal and perinatal deaths* (41,63)<br>• *Tools were not available* (17,19), *especially tools to report and document neonatal death cases* (19)<br>*Systems:*<br>• *Parallel or dual reporting systems in contexts with multiple stakeholders supporting and/or implementing MPDSR systems (e.g., UNHCR and government MPDSR systems)* (63)<br>• *Electronic or digital systems utilized: the electronic JSANDS for the reporting of stillbirths and neonatal deaths* (43–48) *or electronic dashboards to monitor maternal and perinatal deaths reported to the IDSR* (21)<br>• *Lack of follow-up or monitoring and evaluation systems for MPDSR interventions* (14,20,21)
Fidelity: *The degree to which MPDSR and related death review interventions were implemented as intended, according to local, national, or international guidelines or action plans*		
Adherence to implementation processes	*24*	• *Under-reporting of maternal and perinatal deaths* (14,15,19–21,26,27,41,49–51,54,56,59,63), *including the under-reporting of these deaths in transit* (14).<br>• *Only a sample of reported maternal and perinatal deaths reviewed* (14,17–22,41,53–56,63)<br>• *Facility-based reviews and community-based verbal autopsies conducted for all reported deaths in CXB* (61)<br>• *Delays in the reporting of maternal and/or perinatal deaths* (15,19,21,41,51,62,63)<br>• *Delays in reviewing the cases* (16,17,21,41,55,62)<br>• *Limited uptake of recommendations and/or response to address identified issues* (14,17,18,20–22,54,56,60,63)<br>• *Weak reporting and transmission of information related to maternal and perinatal deaths from facilities to national level* (22,54)
Quality of reporting & review	*23*	• *Low quality of available data on maternal and perinatal death cases due to inadequate patient information in facility records* (14,17,18,21,26,41,43,55,56,58,60,63), *inaccessible information in referral facilities/health systems* (17,41,60), *unclassified or misclassified deaths (no cause specified, wrong cause specified, mix of neonatal/stillbirth)* (14,26,50,51,56,59,62,63), *and missing or incomplete reporting using death review forms and tools* (18,20,41,51,55,56,62).<br>• *Variable definitions of stillbirths* (14) *and households* (15) *created issues in data quality.*<br>• *Population movement and distorted household sizes* (15,27) *affect reliability of denominators to calculate mortality rates*<br>• *Divergent systems, interventions, and/or data sources related to maternal and perinatal death, especially between partners or with government systems, impacts the quality of reporting and review of maternal and perinatal deaths* (19,60,63).<br>• *Local adaptations of international tools for reporting and reviewing maternal and perinatal deaths oversimplified data* (14,48,60)<br>• *Paper tools facilitate the manipulation and/or concealment of data related to death cases* (14).<br>• *Unavailability of tools* (17) *and health information systems* (43) *created implementation difficulties.*<br>• *Data dashboards facilitated the monitoring of mortality trends but provided no analysis or information related to death reviews* (21).<br>• *Lack of disaggregation of data extracted from national surveillance or health information systems for populations affected by crises (refugee, IDP, etc.)* (63) *limited MPDSR insights within humanitarian contexts.*<br>• *During death review meetings, a misalignment between identified problems and solutions thwarted impactful response efforts* (14,18).
Implementing actor responsiveness	*16*	*Overall participation:*<br>• *Low health provider participation* (14,17,19,20,41,43,48,56,63)

Construct	# of articles or reports	Summary of findings
		• *Demotivating factors: a lack of buy-in to the value of MPDSR interventions* (17,43,56), *a lack of or inadequacy in the uptake of recommendations and action points identified during reviews to improve services* (14,17,56), *a lack of financial support* (17,48,56,63), *limited or lack of time to participate* (14,43,56), *the noninvolvement of some key staff in review and response activities* (17), *and concerns about potential scrutiny and damage of reputation* (14,19).<br>• *Incentivized MPDSR interventions employing performance-based financing for death reporting and reviews were successful in ensuring adoption and fidelity of MPDSR interventions* (21,56).<br>• *Good participation of actors in community-based approaches* (15,63)<br>• *Good participation (>50% of cases) from family members in maternal death audits was reported in the East and Horn of Africa and Great Lakes Region* (41).<br>• *Limited or no community involvement including a lack of community awareness of system hindered uptake of reporting, review, and response steps of MPDSR interventions* (20,56)<br>*Review committee participation:*<br>• *Limited participation in maternal or perinatal death review meetings* (16,17,20,21,48,56,63)<br>• *Demotivating factors: lack of financial support for participants in the review sessions* (17,48,56,63), *lack of interest in participation by non-clinician actors* (16), *the silencing of voices of participating members during review meetings due to hierarchies* (16), *and a lack of supportive policies for the functionality of review committees* (20,63).<br>• *Issues ensuring confidentiality* (43) *and anonymity* (16,17) *influenced the quality of maternal and perinatal death reviews.*<br>• *JSANDS in Jordan reported active facility committee participation, yet emphasized need for national review committees* (47).<br>*Health facility administration:*<br>• *Limited or no supervision of the implementation of MPDSR interventions by facility administration* (17–21)<br>• *Limited monitoring and evaluation of identified recommendations and action items derived from death reviews (i.e., response) by hospital leadership* (18–20,22).
Penetration: *The integration of MPDSR and related death review interventions within health systems in humanitarian settings*		
Positionality within health system	*9*	• *The institutionalization of MPDSR interventions into health systems varied greatly between humanitarian settings* (14,17,26,47,51,55,56,63)<br>• *In Uganda, MPDSR interventions led by humanitarian partners are integrated into the national MPDSR system* (63)<br>• *Collaboration and coordination across different levels of the health system (i.e., between review committees at facility, subnational, and national levels or between referring facilities) was limited* (17,63).<br>*Strategies for integration:*<br>• *Integration of maternal and near-miss reviews in Palestine* (63)<br>• *Hospital accreditation dependent upon establishment of neonatal death review committees in Jordan* (47)<br>• *Institutionalization of review committees within health system in Cameroon* (53)<br>• *MPDSR interventions linked with QI committees or activities in Nigeria and Afghanistan* (55,56).
Interoperability with other surveillance systems	*10*	• *Many MPDSR interventions leveraged existing surveillance systems for the identification or report of maternal and perinatal deaths including the IDSR* (20,21,54)*, WHO's EWARS in CXB* (27,61–63), *the MESI in Haiti* (42), *and the Maternal and Child Health e-Registry in Palestine* (60).<br>• *CRVS was employed to triangulate reported deaths in Jordan* (46)<br>• *In other humanitarian contexts, CRVS was utilized to lesser extent due to gaps or deficiencies in registration of deaths* (49,63).
Sustainability: *The extent to which MPDSR and related death review interventions are institutionalized within a health system or humanitarian programming*		
Local ownership	*1*	• *Maternal mortality surveillance system established, designed, and implemented by Ministry of Health of Palestine* (60)
Sustained funding streams	*0*	• *No sustained funding streams reported*
Institutionalized capacity	*0*	• *Not reported – training expressed as gap/need across contexts.*
Appropriateness: *Perceived fit or relevance of MPDSR and related death review interventions within humanitarian settings.*		

Construct	***# of articles or reports***	***Summary of findings***
Perceived relevance	*14*	*Relevant:*<br>• *Capacity to understand health system performance, identify gaps in service delivery and mount quality improvement activities to prevent future deaths* (16,22,27,42,50,55,56).<br>• *Potential for change in policy and provider practice due to learning and findings from MPDSR and related death review interventions* (25,47,48)<br>• *Acquisition of data on maternal and perinatal mortality within crisis affected areas and opportunity to understand the magnitude of the crisis, identify marginalized populations, and address disparities in care, especially for refugees and IDPs* (44,48,63)<br>• *Importance in understanding and addressing community and cultural factors influencing health decision-making* (14)<br>• *Community engagement to build trust between communities and humanitarian actors* (27)<br>*Not relevant:*<br>• *MPDSR is not considered a component of humanitarian duty but rather an intervention for development contexts which translates into less attention and interest among humanitarian actors* (63)
Perceived complexity	*4*	• *Complex forms and standardized tools* (14,18)<br>• *Complexity of community-based approaches with high personnel requirements* (27) *and hierarchical reporting and death verification systems* (62)
**Notes:**<br>*Abbreviations: CHW: community health worker; CRVS: Civil registration and vital statistics; CXB: Cox's Bazar; EWARS: Early Warning, Alert, and Response System; IDP: internally displaced persons; IDSR: Integrated disease surveillance and response; JSANDS: Jordan Stillbirth and Neonatal Death Surveillance; MESI: Integrated Monitoring, Evaluation, and Surveillance System; MoH: Ministry of Health; MPDSR: Maternal and perinatal death surveillance and response; QI: quality improvement; TBA: traditional birth attendant; TOT: training of trainers; UNHCR: United Nations High Commissioner for Refugees; WHO: World Health Organization*		

Appendix S1: Search Strategy

1. Pubmed/MEDLINE

Concept 1: MPDSR and related death review interventions

((("maternal death"[mesh] OR ("maternal"[tw] AND "death*"[tw]) OR "perinatal death[mesh]" OR ("perinatal"[tw] AND "death*"[tw]) OR ("peri-natal"[tw] AND "death*"[tw]) OR ("neonatal"[tw] AND "death*"[tw]) OR ("neo-natal"[tw] AND "death*"[tw]) OR ("newborn"[tw] AND "death*"[tw]) OR ((pregnan*[tw] OR pregnancy[mesh]) AND (death*[tw] OR "mortalit*"[tw])) OR "stillbirth"[mesh] OR "stillbirth*"[tw] OR "still birth*"[tw] OR "maternal mortality"[mesh] OR ("maternal"[tw] AND "mortalit*"[tw]) OR "perinatal mortality"[mesh] OR ("perinatal"[tw] AND "mortalit*"[tw]) OR ("peri-natal"[tw] AND "mortalit*"[tw]) OR ("neonatal"[tw] AND "mortalit*"[tw]) OR ("neo-natal"[tw] AND "mortalit*"[tw]) OR ("newborn"[tw] AND "mortalit*"[tw]) OR "fetal death"[mesh] OR "fetal death*"[tw] OR "fetal demise"[tw])) AND

(("clinical audit"[mesh] OR "death audit*"[tw] OR "mortality audit*"[tw] OR "public health surveillance"[mesh] OR "surveillance"[tw] OR "autopsy"[mesh] OR "verbal autops*"[tw] OR "social autops*"[tw] OR "death review*"[tw] OR "mortality review*"[tw] OR "MPDSR"[tw] OR "MDSR"[tw] OR "PDSR"[tw] OR "NDSR"[tw] OR "confidential inquir*"[tw] OR "confidential enquir*"[tw])))

Concept 2: Humanitarian Settings

("refugee camps"[mesh] OR "refugees"[mesh] OR "Relief Work"[Mesh] OR "humanitarian"[tw] OR "crisis*"[tw] OR "conflict*"[tw] OR "emergencies"[mesh] OR "refugee*"[tw] OR "internally displaced"[tw] OR "returnee*"[tw] OR "Disasters"[Mesh] OR Disaster* [tw] OR "Armed Conflicts"[Mesh] OR war[tw] OR wars[tw] OR Afghanistan [tw] OR Bangladesh [tw] OR "Burkina Faso" [tw] OR Burundi [tw] OR Cameroon [tw] OR "Central African Republic" [tw] OR Chad [tw] OR Colombia [tw] OR "Democratic Republic of the Congo" [tw] OR Ethiopia [tw] OR Haiti [tw] OR Iraq [tw] OR Jordan [tw] OR Kenya [tw] OR Libya [tw] OR Mali [tw] OR Mozambique [tw] OR Myanmar [tw] OR Niger [tw] OR Nigeria [tw] OR oPT [tw] OR "occupied Palestinian territor*" [tw] OR Rwanda [tw] OR Somalia [tw] OR "South Sudan" [tw] OR Sudan [tw] OR Syria [tw] OR Tanzania [tw] OR Ukraine [tw] OR Uganda [tw] OR Venezuela [tw] OR Yemen [tw])

2. SCOPUS

Concept 1: MPDSR and related death review interventions

((TITLE-ABS-KEY(maternal PRE/4 death) OR TITLE-ABS-KEY(perinatal PRE/4 death) OR TITLE-ABS-KEY(neonatal PRE/4 death) OR TITLE-ABS-KEY(newborn PRE/4 death) OR TITLE-ABS-KEY(pregnan* PRE/4 death) OR TITLE-ABS-KEY(pregnan* W/4 mortalit*) OR TITLE-ABS-KEY(stillbirth) OR TITLE-ABS-KEY("still birth") OR TITLE-ABS-KEY(maternal PRE/4 mortalit*) OR TITLE-ABS-KEY(perinatal PRE/4 mortalit*) OR TITLE-ABS-KEY(neonatal PRE/4 mortalit*) OR TITLE-ABS-KEY(newborn PRE/4 mortalit*) OR TITLE-ABS-KEY(fetal PRE/4 death) OR TITLE-ABS-KEY("fetal demise")) AND

(TITLE-ABS-KEY("death audit") OR TITLE-ABS-KEY("mortality audit") OR TITLE-ABS-KEY(surveillance) OR TITLE-ABS-KEY("verbal autops*") OR TITLE-ABS-KEY("social autops*") OR TITLE-ABS-KEY("death review") OR TITLE-ABS-KEY("mortality review") OR TITLE-ABS-KEY(MPDSR) OR TITLE-ABS-KEY(MDSR) OR TITLE-ABS-KEY(PDSR) OR TITLE-ABS-KEY(NDSR) OR TITLE-ABS-KEY("confidential inquir*") OR TITLE-ABS-KEY("confidential enquir*")))

Concept 2: Humanitarian Settings

(TITLE-ABS-KEY(refugee) OR TITLE-ABS-KEY("Relief Work") OR TITLE-ABS-KEY(humanitarian) OR TITLE-ABS-KEY(crisis) OR TITLE-ABS-KEY(conflict) OR TITLE-ABS-KEY(internally PRE/4 displaced) OR TITLE-ABS-KEY(returnee) OR TITLE-ABS-KEY(disaster) OR TITLE-ABS-KEY(war) OR TITLE-ABS-KEY(Afghanistan) OR TITLE-ABS-KEY(Bangladesh) OR TITLE-ABS-KEY({Burkina Faso}) OR TITLE-ABS-KEY(Burundi) OR TITLE-ABS-KEY(Cameroon) OR TITLE-ABS-KEY({Central African Republic}) OR TITLE-ABS-KEY(Chad) OR TITLE-ABS-KEY(Colombia) OR TITLE-ABS-KEY({Democratic Republic of the Congo}) OR TITLE-ABS-KEY(Ethiopia) OR TITLE-ABS-KEY(Haiti) OR TITLE-ABS-KEY(Iraq) OR TITLE-ABS-KEY(Jordan) OR TITLE-ABS-KEY(Kenya) OR TITLE-ABS-KEY(Libya) OR TITLE-ABS-KEY(Mali) OR TITLE-ABS-KEY(Mozambique) OR TITLE-ABS-KEY(Myanmar) OR TITLE-ABS-KEY(Niger) OR TITLE-ABS-KEY(Nigeria) OR TITLE-ABS-KEY("oPT") OR TITLE-ABS-KEY("occupied Palestinian territor*") OR TITLE-ABS-KEY(Pakistan) OR TITLE-ABS-KEY(Rwanda) OR TITLE-ABS-KEY(Somalia) OR TITLE-ABS-KEY(Sudan) OR TITLE-ABS-KEY(Syria) OR TITLE-ABS-KEY(Tanzania) OR TITLE-ABS-KEY(Ukraine) OR TITLE-ABS-KEY(Uganda) OR TITLE-ABS-KEY(Venezuela) OR TITLE-ABS-KEY(Yemen))

3. EMBASE

Concept 1: MPDSR and related death review interventions

(('perinatal death'/exp OR 'maternal death'/exp OR 'newborn death'/exp OR 'stillbirth'/exp OR 'maternal mortality'/exp OR 'perinatal mortality'/exp OR 'newborn mortality'/exp OR 'fetal demise'/exp OR 'fetus mortality'/exp OR (maternal OR perinatal OR neonatal OR newborn OR fetal) NEXT/4 (death* OR mortalit*) OR pregnan* NEAR/4 (death* OR mortalit*) OR stillbirth* OR 'still birth*' OR 'fetal demise') AND

('clinical audit'/exp OR 'epidemiological surveillance'/exp OR 'autopsy'/exp OR 'verbal autopsy'/exp OR 'death audit*' OR 'mortality audit*' OR surveillance OR 'verbal autops*' OR 'social autops*' OR 'death review*' OR 'mortality review*' OR 'MPDSR' OR 'MDSR' OR 'PDSR' OR 'NDSR' OR 'confidential inquir*' OR 'confidential enquir*'))

Concept 2: Humanitarian Settings

('disaster'/exp OR 'forced migrant'/exp OR 'displacement (people)'/exp OR 'refugee camp'/exp OR 'relief work'/exp OR 'disaster management'/exp OR 'war'/exp OR 'warfare/exp' OR 'humanitarian intervention'/exp OR refugee* OR 'Relief Work' OR humanitarian OR crisis OR conflict* OR internally NEXT/4 displaced OR returnee* OR disaster* OR war OR wars OR afghanistan:ab,ti,kw,de OR bangladesh:ab,ti,kw,de OR 'burkina faso':ab,ti,kw,de OR burundi:ab,ti,kw,de OR cameroon:ab,ti,kw,de OR 'central african republic':ab,ti,kw,de OR chad:ab,ti,kw,de OR colombia:ab,ti,kw,de OR 'democratic republic of the congo':ab,ti,kw OR ethiopia:ab,ti,kw,de OR haiti:ab,ti,kw,de OR iraq:ab,ti,kw,de OR jordan:ab,ti,kw,de OR kenya:ab,ti,kw,de OR libya:ab,ti,kw,de OR mali:ab,ti,kw,de OR mozambique:ab,ti,kw,de OR myanmar:ab,ti,kw,de OR niger:ab,ti,kw,de OR nigeria:ab,ti,kw,de OR 'opt':ab,ti,kw OR 'occupied palestinian territor*':ab,ti,kw OR rwanda:ab,ti,kw,de OR somalia:ab,ti,kw,de OR 'south sudan':ab,ti,kw,de OR sudan:ab,ti,kw,de OR syria:ab,ti,kw,de OR tanzania:ab,ti,kw,de OR ukraine:ab,ti,kw,de OR uganda:ab,ti,kw,de OR venezuela:ab,ti,kw,de OR yemen:ab,ti,kw,de OR 'palestine'/de OR 'democratic republic congo'/de)

4. Grey literature search

Requests to key informants at:

- Global and regional offices of UNHCR, UNFPA, UNICEF, UNRWA, and WHO

- Key organizations working in humanitarian contexts including International Rescue Committee, CARE, Save the Children, CDC, Jhpiego, etc.
- Governmental entities in collaboration with UN or humanitarian agencies
- Technical working groups focused on supporting implementation of MPDSR
- Inter-agency working group for reproductive health in emergencies

Electronic/internet searches on:

- Humanitarian web-portals (e.g., ReliefWeb and Humanitarianresponse.info)
